# The Ratio Optimization and Strength Mechanism of Composite Cementitious Material with Low-Quality Fly Ash

**DOI:** 10.3390/gels8030151

**Published:** 2022-03-01

**Authors:** Xiaobing Yang, Zepeng Yan, Shenghua Yin, Qian Gao, Weiguang Li

**Affiliations:** 1 School of Civil and Resource Engineering, University of Science and Technology Beijing, Beijing 100083, China; yangxiaobing@ustb.edu.cn (X.Y.); ustb_ysh@163.com (S.Y.); gaoqian@ces.ustb.edu.cn (Q.G.); 2 State Key Laboratory of Mineral Processing, Beijing 102628, China; liweiguang@bgrimm.com

**Keywords:** filling mining, cementitious materials, low calcium fly ash, proportional optimization, strength

## Abstract

To resolve the limited large-scale methods of disposal of low calcium fly ash with poor activity, based on the double excitation principle, clinker and desulfurized gypsum are used as alkali/salt activators to activate fly ash and slag, avoiding the inconvenience of strong alkali activating fly ash in industry. Firstly, the strength test of a filling body with multiple ratio composite cementing material is carried out, and the weight coefficient of each material to strength is analyzed by grey correlation degree. The composition of the hydration products, microstructure, and pore structure of the filling body was analyzed by X-ray diffractometer, scanning electron microscope, thermogravimetric test, and mercury compression test. The strength mechanism of the cemented body was confirmed. The results show that cemented backfill prepared by composite cementitious material, which contained high content and low-quality fly ash, can meet the strength requirements of subsequent backfill in a mine. The degree of composite cementitious material influence on 7 d strength is slag > desulfurized gypsum > fly ash > clinker; the degree of influence on 28 d strength is: fly ash > slag > desulfurized gypsum > clinker. The main hydration products of the composite cementable material with high content low-quality fly ash are C–S–H gel and ettringite, and the unreacted fly ash particles can still be seen at 28 d. As the curing age grows, the difference in the number of hydration products under different proportioning conditions has a weaker effect on the strength, while the influence of raw materials and product morphology on the pore structure determines the development trend of the strength. Therefore, the threshold pore size can be used to characterize the strength advantages and disadvantages reasonably.

## 1. Introduction

Fly ash is a byproduct of coal-fired power plants. The fly ash from most power plants in China has low quality, which is generally grade III or off grade, with low calcium content [1]. The main source of activity of poor quality fly ash is vitreous, which requires large internal energy to be excited and presents a low activity state at room temperature. Moreover, the rate and degree of early hydration reaction are extremely low, so there are few ways to utilize fly ash, which is substantially accumulated and can cause serious environmental issues [2,3]. Analysis of its chemical composition finds that various components enable its usage as a geopolymer to replace cement in concretes [4]. The cementitious properties of fly ash are attributable to a combination of three effects: pozzolanic reaction effect, micro-aggregate effect, and particle morphology effect. Among them, the pozzolanic reaction is chemical, while the other two effects are physical [5,6,7].

Extensive existing studies [8,9,10] have shown that slag addition can prominently improve the mechanical properties of fly ash geopolymer pastes, mortars, and concretes. Yang et al. [11] used NaOH and sodium silicate to activate a fly ash–slag powder composite-based geopolymer, and investigated the effects of slag powder content on the macro mechanical properties and microstructure of fly ash geopolymer mortars at different curing temperatures. However, these studies all use strong alkali solutions to stimulate the activity of fly ash and slag, which is industrially inapplicable (due to limitations from production equipment and conditions), and easily results in alkali–aggregate reaction. Lv et al. [12] activated a fly ash-based geopolymer by using sodium sulfate (instead of strong alkali) combined with sodium silicate, and explored how sulfate content and water–cement ratio variation affected the strength and microstructure. However, given the lack of calcium ions in this activation system, the hydration product was sodium aluminosilicate hydrate (N–A–S–H), which was less dense than calcium silicate hydrate (C–S–H), and was, thus, detrimental to improving the cement strength [13]. By adding desulfurized gypsum into the bottom slag and fly ash, Boonserm Kornkanok et al. [14] prepared geopolymers with NaOH and sodium silicate activators. Research has found that adding 5–10% of desulfurized gypsum can enhance the strength of geopolymers. This is primarily attributed to the presence of SO_4_^2−^, which leads to the increase in the Al3+ leached from bottom ash and the formation of additional calcium silicate hydrate. Nevertheless, 10–15% of desulfurized gypsum will produce anhydrous thenardite to induce micro-cracks, thereby weakening the system strength. Despite the sulfate incorporation in the foregoing research, anhydrous thenardite and N–A–S–H were produced due to the presence of Na^+^, which was detrimental to enhancing the system strength. Hence, it is imperative to adopt an appropriate activator, and to investigate its activation mechanism and effects on the hydration products and microstructure of cements.

In addition to unfavorable properties such as the need for strong alkali activation, Zhao [15] summarized the durability of fly ash-based geopolymers, which had worse resistances to carbonation, freeze–thaw, and weathering than Portland cement concretes. Thus, their broad application in construction engineering still has a long way to go. Meanwhile, mine backfilling requires considerably lower backfill strength and durability than concretes [16], and is strongly adaptable to raw materials, which offers an opportunity for the utilization of low-quality fly ash. Sun et al. [17] fabricated a coal gangue conglomerate paste filler by using activated coal gangue supplemented by fly ash and other materials. Apart from better durability, its physical and mechanical properties can all conform to the national cement standards. Wu et al. [18] measured the hydration heats of filler materials with different proportions of fly ash, and predicted the elevation in filling body temperature caused by 7-d accumulated hydration heat without considering the heat exchange with the outside. However, there have been scarce studies concerning the proportion optimization of cementitious composites, or the curing characteristics/microstructure of cements, and the conclusions have focused on the correlation of slag content with strength and setting time [19,20,21].

Based on the dual activation theory, this paper activated a fly ash–slag cementitious composite by using clinker as the alkali activator and desulfurized gypsum as the salt activator. Grey correlation degree was used to analyze the weighting coefficients of various material ratios against strength, while X-ray diffractometry (XRD), scanning electron microscopy (SEM), thermogravimetric analysis (TGA), and mercury intrusion porosimetry (MIP) were employed to analyze the hydration product composition, microstructure, and pore structure of the cementitious composite. The hydration mechanism of high-volume low-quality fly ash cementitious composite was revealed, and the feasibility of developing novel cementitious fillers was discussed.

## 2. Results and Discussion

### 2.1. Effect of Active Ingredients on Strength

#### 2.1.1. Orthogonal Results Analysis

**Figure 1:**
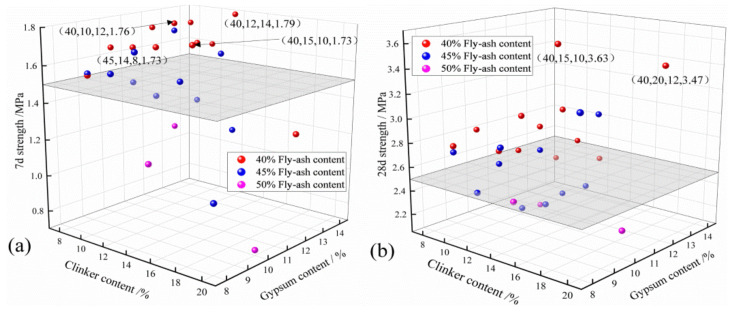
Composite cementitious material filling body strength test result: (**a**) 7 d, (**b**) 28 d.

Figure 1 displays the strength test results of filler cement for the cementitious composite. As is clear, the 7-d and 28-d strengths varied prominently with the fly ash content, which decreased with the increasing fly ash content overall. After comprehensively considering the 7-d and 28-d strengths, the optimal proportion could be derived from the test results as follows: fly ash content 40%, clinker content 15%, desulfurized gypsum content 10%, 7-d strength 1.73 MPa, and 28-d strength 3.63 MPa.

**Figure 2:**
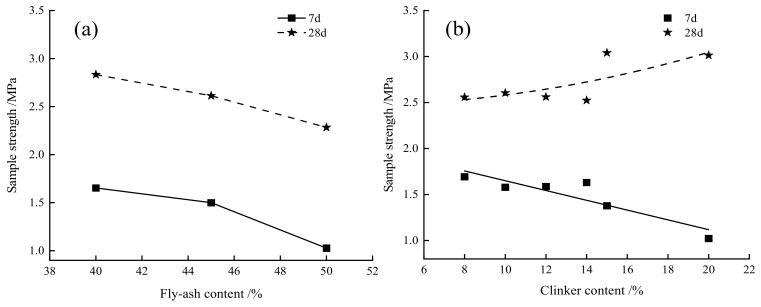

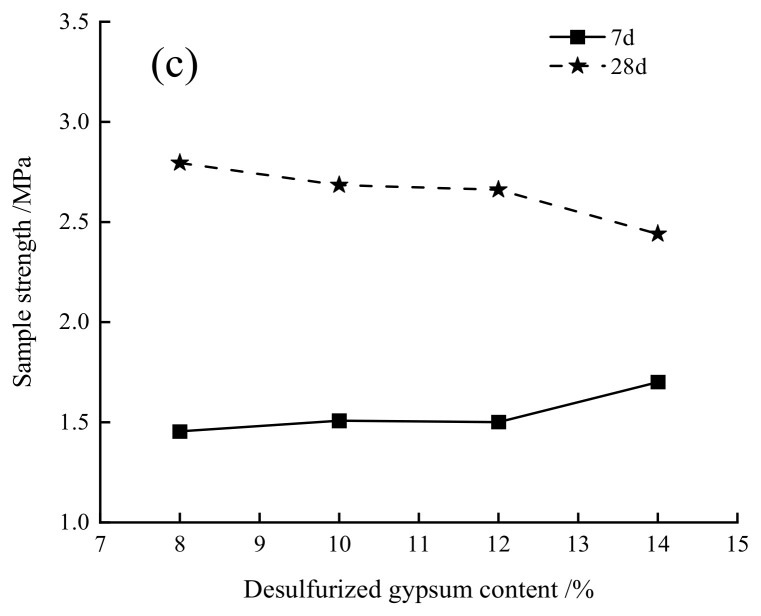
The relationship between the amount of each cementitious material component and the filling body strength: (**a**) fly ash, (**b**) clinker, (**c**) desulfurized gypsum.

Figure 2 describes the range analysis of the test results. Clearly, the 7-d and 28-d strengths tended to decrease progressively with the increasing fly ash content. As for clinker, it had little effect on the strength at contents below 14%. When the clinker content exceeded 14%, the 7-d strength showed a downward trend, while the 28-d strength showed an upward trend. This was primarily attributed to the dissolution of slag vitreous structure by the alkaline environment generated in the later hydration stage at high clinker contents, which resulted in the release of substantial Ca^2+^, active SiO_2_, and Al_2_O_3_ [22,23]. With the increasing content of desulfurized gypsum, the 7-d strength showed an upward trend, while the 28-d strength showed a downward trend. This was primarily attributed to the fast dissolution of gypsum in the early hydration stage, which facilitated the rapid formation of ettringite (Aft) [24]. Meanwhile, the incorporation of gypsum inhibited the conversion of active Al_2_O_3_ produced by slag hydration into C_4_AH_13_, thereby promoting the reaction of active Al_2_O_3_ with gypsum to form AFt while consuming partial Ca(OH)_2_ [25,26].

#### 2.1.2. Grey Correlation Analysis

The influencing weights of various cementitious composite proportions on the cement strength were determined by grey correlation analysis, which evaluated the degree of correlation between things (factors) based on the similarity level of thing (factor) sequence curve geometries through a quantitative approach [27,28]. The specific computational process is as follows.

A matrix (1) was constructed as shown below by regarding the 7-d (*X*_0_) and 28-d (*X*_1_) cement strengths as the reference sequence, and the fly ash content (*X*_2_), clinker content (*X*_3_), desulfurized gypsum content (*X*_4_), and slag content (*X*_5_) as the comparative sequence, where *n* = 5, *m* = 27.

(1) $$\left(X_{0},X_{1},\cdots ,X_{n}\right)=\left[\begin{array}{cccc} X_{0}\left(1\right) & X_{1}\left(1\right) & \cdots & X_{n}\left(1\right)\\ X_{0}\left(2\right) & X_{1}\left(2\right) & \cdots & X_{n}\left(2\right)\\ \vdots & \vdots & & \vdots \\ X_{0}\left(m\right) & X_{1}\left(m\right) & \cdots & X_{n}\left(m\right) \end{array}\right]$$

Given the differing physical significances of the factors in the above matrix, the data dimensionalities were also inconsistent. For the convenience of comparison, the matrix data were made dimensionless initially by Formula (2) during the grey correlation analysis, thereby obtaining a new data matrix [29]. Further, the correlation coefficients were calculated by Formula (3).

(2) $$\begin{array}{c} X_{i}^{\prime }\left(k\right)=\frac{X_{i}\left(k\right)}{\frac{1}{m}\sum_{k=1}^{m}X_{i}\left(k\right)}\\ \left(i=0,1,2,\cdots ,n;k=1,2,\cdots ,m\right) \end{array}$$

(3) $$\begin{array}{c} L_{i}\left(k\right)=\frac{\underset{n}{\min}\;\underset{m}{\min}\left|X_{{}_{i}}^{\prime }\left(k\right)-X_{{}_{0}}^{\prime }\left(k\right)\right|+\rho \;\underset{n}{\max}\;\underset{m}{\max}\left|X_{{}_{i}}^{\prime }\left(k\right)-X_{{}_{0}}^{\prime }\left(k\right)\right|}{\left|X_{{}_{i}}^{\prime }\left(k\right)-X_{{}_{0}}^{\prime }\left(k\right)\right|+\rho \;\underset{n}{\max}\;\underset{m}{\max}\left|X_{{}_{i}}^{\prime }\left(k\right)-X_{{}_{0}}^{\prime }\left(k\right)\right|}\\ \left(i=0,1,2,\cdots ,n;k=1,2,\cdots ,m\right) \end{array}$$

In the case of 7-d strength *X*_0_ (when calculating 28-d strength, *X*_0_ was replaced with *X*_1_), *L_i_*(*k*) in the above formula represents the relative difference, i.e., correlation coefficient, between the comparative sequence *X_i_* and the reference sequence *X*_0_ at time *k*. To distinguish coefficients, *ρ* was designed to weaken the distortion caused by excessively large maximum absolute difference, and to improve the significance of differences between correlation coefficients, whose value was generally 0–1 [30,31]. In this study, *ρ* was set at 0.5.

The correlation degree between reference and comparative sequences was calculated using the mean correlation coefficients of two comparative sequences at each time [32]. The specific computational procedure is shown in formula (4).

(4) $$r_{i}=\frac{1}{m}\sum_{k=1}^{m}L_{i}\left(k\right)$$

Following the above steps, the correlation degrees of 7-d and 28-d strengths with four cementitious material components (fly ash, clinker, desulfurized gypsum, and slag) were calculated as shown in Table 1. Clearly, the influencing weights on the 7-d strength were: slag > desulfurized gypsum > fly ash > clinker, while the influencing weights on the 28-d strength were: fly ash > slag > desulfurized gypsum > clinker. In the early stage of hydration reaction, the slag and desulfurized gypsum had the greatest influences, which were attributed to the AFt produced by further reaction between desulfurized gypsum and slag hydration products. Cement clinker had the weakest influences on both the 7-d and 28-d strengths. According to the range analysis of the previous orthogonal test, the cement clinker had insignificant influence on the 7-d strength, while enhanced the 28-d strength. Fly ash significantly reduced the 7-d and 28-d strengths, and such influence was most significant on the 28-d strength.

It is clear from the grey correlation analysis that slag, as the main active constituent, produced the most significant effect on the cement strength. According to the literature review, the effect of fly ash is second only to that of slag, followed by desulfurized gypsum and clinker. Next, the cement strength mechanism of fly ash cementitious composite was carried out.

### 2.2. Effect of Fly Ash Content on Hydration Product

#### 2.2.1. XRD

**Figure 3:**
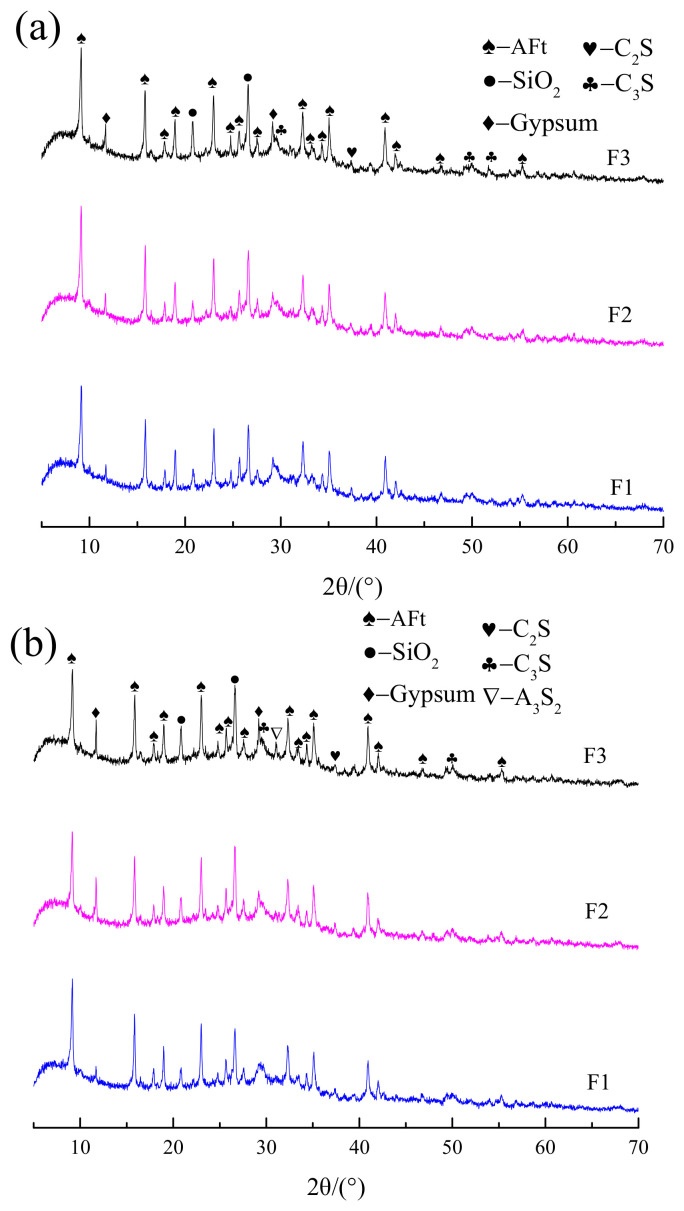
XRD spectra of F1, F2, and F3 groups at 7 d and 28 d: (**a**) 7 d, (**b**) 28 d.

Figure 3 illustrates the XRD pattern of the 7-d and 28-d hydration products of F1, F2, and F3 groups. The JCPDS database was used to analyze the XRD patterns, and it was found that the primary hydration product of the fly ash cementitious material at 7-d and 28-d ages was AFt, while the amorphous C–S–H gel could hardly be reflected on XRD. It is noteworthy that the silica peaks appeared on the XRD spectra at both 7-d and 28-d ages, which was ascribed to the unhydrated fly ash. Compared to the inert silica peaks, the AFt peak values at 28 d in the F1 group all increased significantly more than those at 7 d of age. Meanwhile, the hydration products in the F3 group increased comparatively mildly. On the contrary, the gypsum peaks increased greatly at 28 d than those at 7 d of age, indicating a higher degree of hydration reaction in the F1 group than that in the F3 group. Moreover, compared to the early hydration stage in the F3 group, the amount of dihydrate gypsum involved in the hydration reaction decreased in the late stage. This was primarily attributed to the very slow secondary reaction of fly ash even after 90 d. At the 7-d age, the diffraction peaks of the F1, F2, and F3 groups were basically consistent, which were hardly distinguishable. They were also basically consistent at the 28-d age, except for higher peaks of silica and gypsum in the F3 group, suggesting the presence of more unhydrated active fly ash substances in this group.

#### 2.2.2. TG/DTG

**Figure 4:**
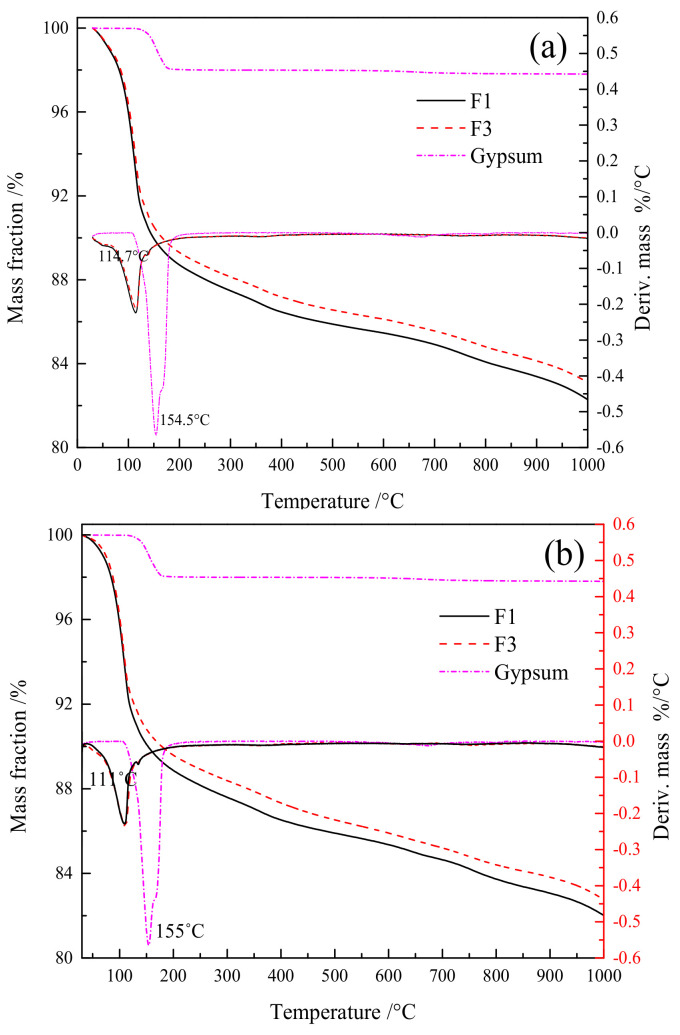
TG/DTG curve of F1 and F3 groups at 7 d and 28 d: (**a**) 7 d, (**b**) 28 d.

**Figure 5:**
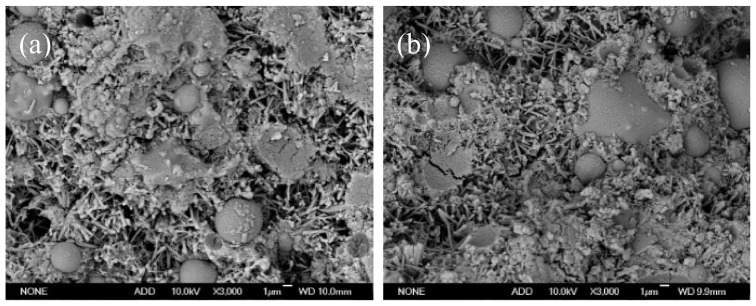
SEM photo of F1 and F3 groups with high fly ash content at 7 d: (**a**) F1, (**b**) F3.

Figure 4 depicts the 7-d and 28-d TG/DTG diagrams for the F1 and F3 groups, and desulfurized gypsum. Among them, the heating range during the test is 30~1000 °C, and the mass loss is converted according to the content. Figure 5 shows that the TG/DTG curves of the F1 and F3 groups at different ages all had a rather obvious peak near 110 °C. The heat loss in this interval was caused primarily by the dehydration reaction of such products as hydrated calcium aluminosilicate gel and ettringite (AFT). Meanwhile, the dehydration of gypsum occurred at around 154 °C. There was little mass loss in other temperature ranges, suggesting that the major hydration products of fly ash cementitious material were the gel and AFt.

According to Figure 4, the mass losses of the F1 and F3 were basically identical at around 110 °C, indicating fundamentally resembling early hydration intensities of the two materials, with the F1 group exhibiting a slightly larger amount of dehydration. After 28 d, the dehydration mass loss of the F1 group was larger as well, indicating the presence of more hydration products such as gel and AFt in this group, which explains the reason for the higher filler strength at this proportion to some extent.

### 2.3. Effect of Fly Ash Content on Microstructure

**Figure 6:**
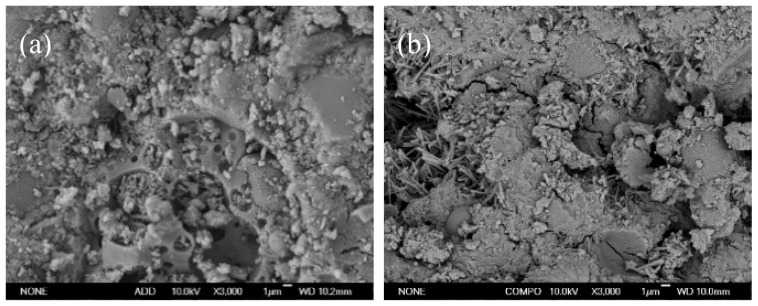
SEM photo of F1 and F3 groups at 28 d: (**a**) F1, (**b**) F3.

Figure 5 and Figure 6 display the SEM micrographs of the fly ash cementitious materials for the F1 and F3 groups. As is clear, substantial rod-shaped AFt and amorphous clustered C–S–H gel could be observed in the 7 d and 28 d hydration products of the two cementitious materials. From the 7 d SEM micrograph, some spherical particles were also observable, which were unhydrated fly ash. The 7 d SEM micrographs differed insignificantly between the two types of materials, indicating the presence of slow hydration and low strength problems with them in the early stage. However, after 28 d, a relatively dense cement microstructure had been formed in the F1 group, while the set cement in the F3 group was not quite dense, with the appearance of some pores and gypsum. Moreover, some unhydrated spherical fly ash was found, which explained the reason for the higher strength of test filler blocks in the F1 group from a microscopic perspective.

### 2.4. Effect of Fly Ash Content on Pore Characteristics

#### 2.4.1. Pore Structure

The filler pores were caused by incomplete compaction or residual air. Some expansive substances in the cementitious material would either increase or decrease the pores. Through the pore development analysis of fillers, the intrinsic cause of strength changes in the fly ash cementitious material could be clarified. The MIP technique could effectively measure the pore distribution and characteristics of test blocks. According to extensive research experiments, the pore sizes can be classified into four levels [33,34]: harmless pores (<20 nm), slightly harmful pores (20–50 nm), harmful pores (50–200 nm), and severely harmful pores (>200 nm). Such classification of pores can relate certain macroscopic properties of cement to the distribution of pores.

**Figure 7:**
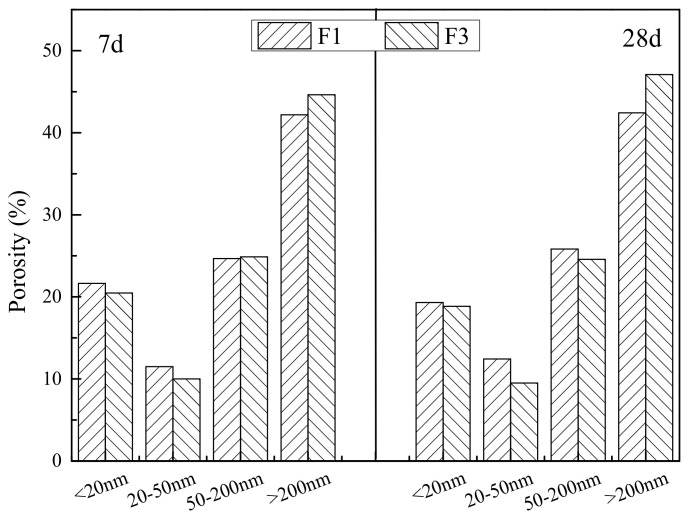
Pore structure parameters of filler at different ages of F1 and F3 groups.

**Figure 8:**
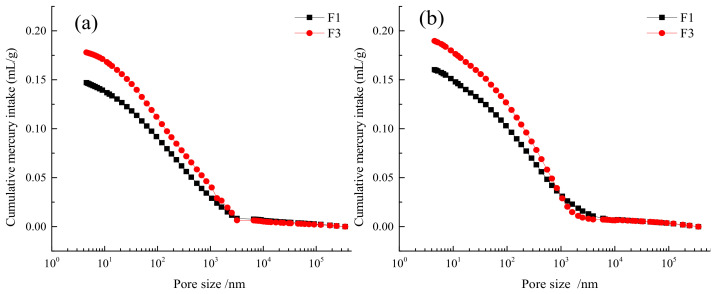
Relationship between cumulative mercury compression and pore size of the filler at different maintenance ages of F1 and F3 groups with high fly ash content: (**a**) 7 d, (**b**) 28 d.

**Figure 9:**
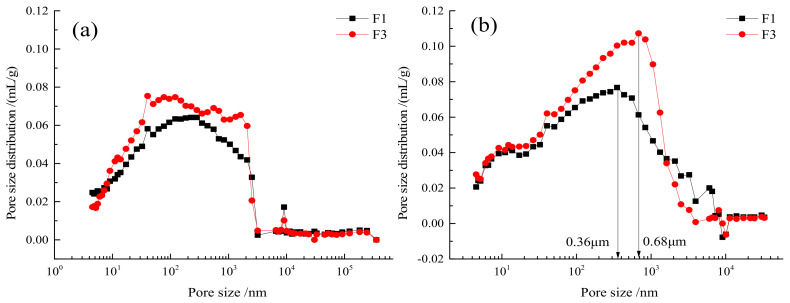
Relationship between differential mercury compression and pore size of the filler at different maintenance ages of F1 and F3 groups with high fly ash content: (**a**) 7 d, (**b**) 28 d.

**Figure 10:**
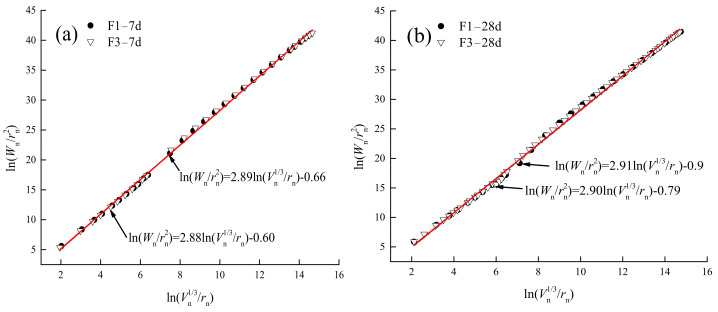
Scatter diagram of slurry pore analysis model for F1 and F3 gelled materials at 7 d and 28 d: (**a**) 7 d, (**b**) 28 d.

The pore structures of the fly ash cementitious filler blocks for the F1 and F3 groups were analyzed by MIP. Table 2 and Figure 7 present the characteristic pore structure parameters of the two types of materials. The correlation between cumulative mercury intrusion volume and pore size is described in Figure 8, whereas the correlation between differential mercury intrusion volume and pore size is illustrated in Figure 9. As is clear, the predominant pores of the two cementitious materials were sorted as: severely harmful pores > harmful pores > harmless pores > slightly harmful pores. At the 7-d and 28-d ages, the porosities of the F1 group were all smaller than those of the F3 group. Nonetheless, the porosities in both groups increased with growing age, which was attributed to the use of expansive AFt as the hydration product. With growing age, the number of harmful pores in the F3 group increased significantly, which was associated with the damage of the full tailings aggregate skeleton by the excessive addition of fly ash. As displayed in Figure 8, the cumulative mercury intrusion volumes in the F3 group were all higher than those in the F1 group at the 7-d and 28-d ages, indicating larger porosities in the F3 group than in the F1 group at both ages. The pore corresponding to the peak on the curve in Figure 10 was the most probable pore, i.e., the pore with the greatest probability of occurrence. It is clear from Figure 8 that the most probable pore size at 28 d was 0.36 μm for the F1 group, while reaching 0.68 μm for the F3 group, with a difference of nearly 2 times. This suggests that the excessive content of fly ash leads to enlarged cement pores and loosened aggregate skeleton, thereby resulting in the decreased strength of the filler cements.

#### 2.4.2. Pore Morphology

The MIP technique has been used to characterize the surface irregularities of many porous solids, such as coal, coke, activated carbon, porous alumina, silica gel, concrete, and building stones [35]. Zhang et al. [36] developed a correlation model between MIP data and pore surface fractal dimension based on thermodynamic analysis, as shown in Formula (5)

(5) $$\ln\left(W_{n}\right)=\ln\left(Q_{n}\right)+C$$

where *W*_n_ denotes the cumulative surface energy of liquid mercury inside pores at the nth intrusion stage, *C* represents the product of liquid mercury surface tension and contact angle cosine, and *Q_n_* stands for the function of pore radius and pore volume at the nth intrusion stage, as shown in Formula (6)

(6) $$Q_{n}=r_{n}^{2-D_{s}}V_{n}^{D_{S}/3}$$

Formula (5) can be modified into a form of (7), in order to eliminate the trial and error and iteration processes. The MIP data were fitted according to (7), thereby deriving the pore fractal model scatter diagrams of the 7-d and 28-d slurries with different fly ash contents and ages. From Figure 10, it is clear that the age and fly ash content basically exerted no effect on the surface roughness of slurry pores, only causing the expansion of pore structures. This suggests that the excess fly ash played a physical filling role.

(7) $$\ln\left(\frac{W_{n}}{r_{n}^{2}}\right)=D_{S}\ln\left(\frac{V_{n}^{1/3}}{r_{n}}\right)+C$$

## 3. Conclusions

1. The proportion of low-quality fly ash-based cementitious composite is identified, whose cement strengths are *R*_7_ = 1.73 MPa and *R*_28_ = 3.63 MPa, thus conforming to the mine filling requirements.
2. The grey correlation analysis revealed that the components with the greatest influence on 7 d and 28 d strength were slag and fly ash, respectively, and it was conjectured that the effect of fly ash on strength was mainly from the hydration reaction and micro-aggregate effect.
3. Although fly ash damages the early strength, it has a gain effect on the long-term strength, which is mainly caused by the secondary hydration reaction of fly ash. Moreover, it was observed that excessive fly ash resulted in long-term strength deterioration, which may be caused by poor skeleton structure.
4. The hydration reaction degree of the cementitious materials prepared with a high-volume of low-quality fly ash is low, and the difference in hydration products does not contribute much to the strength, while the strength difference of the cementitious body benefits from the change in pore structure brought by different ratios of cementitious materials.

## 4. Materials and Methods

### 4.1. Materials

The fly ash and desulfurized gypsum in the cementitious filler material were collected from Dananhu Power Station in Hami, Xinjiang. The main difference between desulfurization gypsum and natural gypsum lies in the different physical states: natural gypsum is in the form of lumps stuck together, while desulfurization gypsum exists as individual crystalline particles.

The fine slag powder was produced by Daan Special Steel in Xinjiang. The original cement clinker was procured locally in Hami and ground to certain fineness with a small indoor mill. Table 3 lists the chemical compositions of various cementitious material constituents. As is clear, the fly ash only contained 2.05% CaO, which was a low-quality low calcium pozzolanic material. The slag was moderately active basic slag [37] with a basicity factor of 1.19 > 1 and a mass factor of 1.72 > 1.6.

**Figure 11:**
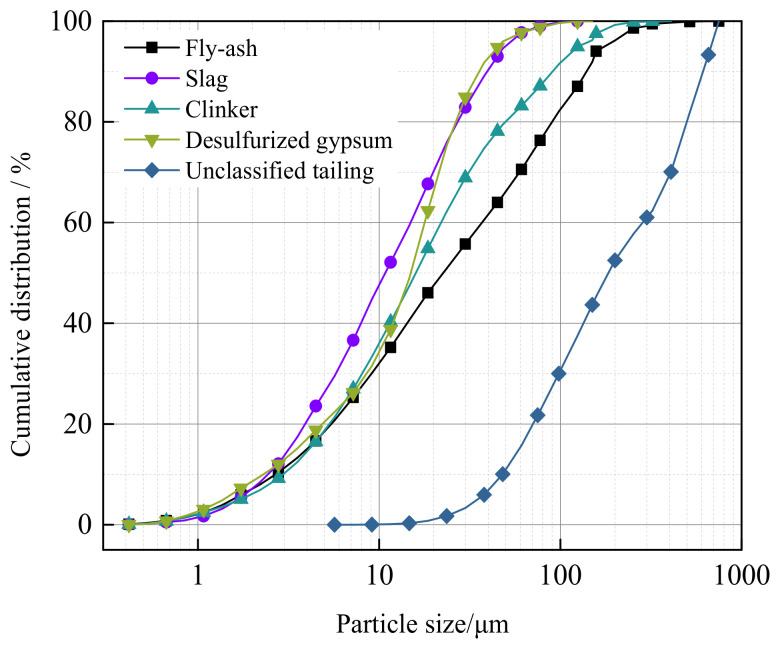
Particle size distribution curve of cementitious material for each component and unclassified tailing.

The filler aggregate was full tailings from the Huangtupo copper–zinc mine. The cumulative particle size distributions of cementitious material and full tailings were measured via LS-POP(9) laser particle size analyzer, as shown in Figure 11. The tailings had relatively coarse particles, with an unevenness coefficient *C*_c_ of slightly greater than 5, indicating narrow size distribution of the tailings particles [38]. Moreover, a curvature coefficient of *C_u_* < 1 indicates lack of medium particle sizes (48.04–285.35 μm). Meanwhile, the fly ash was grade III, with a fineness of 37% and *d*_60_ = 37.04 μm, which could improve the full tailings gradation to some extent.

### 4.2. Experimental Procedures

Conforming to the filling design parameters of mining industry, the strength test of filled cement was carried out by using full tailings as the filler aggregate, and fly ash, desulfurized gypsum, cement clinker, and slag as the cementitious composite. Table 4 describes the test protocol, where a total of 27 groups were set up. The cement–mortar ratio was 1:4 and the slurry concentration was 74%. A standard triple mold (7.07 × 7.07 × 7.07 cm) was used to prepare the test filler blocks. After curing to the age in a YH40-B curing box at 22 ± 1 °C with a humidity > 95%, the uniaxial compressive strengths of the test blocks were measured.

Additionally, through XRD, TG/DTG, SEM, and MIP, the hydration mechanism of the low-quality fly ash cementitious composite was deeply analyzed. The test protocol was as follows: the clinker content was fixed at 15%, the gypsum content was fixed at 10%, the fly ash contents were, respectively, 40%, 45%, and 50%, while the rest was slag. The test blocks were numbered F1, F2, and F3. Pure slurry blocks (20 × 20 × 20 mm) were prepared according to a water–mortar ratio of 0.40. After 1 d of standard curing, the blocks were removed from mold and then wrapped with films, followed by further curing to the specified age under standard conditions. The hydration products of the samples were measured with Ultima IV XRD system and Q600 TGA–differential scanning calorimeter (DSC). The JSM-6700F SEM system was utilized to observe the microstructure of the cementitious slurry under high vacuum conditions, while POREMASTER-33G MIP system was used to determine the porosity characteristics of cured cement.

## Figures and Tables

**Table 1:** Correlation between the age strength and the components of the cementitious material.

Curing Age	Influencing Factors	Fly Ash	Cement Clinker	Desulfurization Gypsum	Slag
7 d	Correlation	0.7839	0.7038	0.7907	0.8891
	Correlation order	3	4	2	1
28 d	Correlation	0.7551	0.6795	0.6913	0.7531
	Correlation order	1	4	3	2

**Table 2:** Pore structure parameters of slurry in different curing ages.

Curing Age	Number	Porosity/%	Pore Diameter Distribution/%			
			<20 nm	20–50 nm	50–200 nm	>200 nm
7 d	F1	22.1	21.65	11.50	24.67	42.18
	F3	27.2	19.31	12.43	25.83	42.42
28 d	F1	25.0	20.47	10.01	24.88	44.63
	F3	29.4	18.84	9.51	24.57	47.08

**Table 3:** The composition of cementitious material for each component.

Materials	SiO_2_	Al_2_O_3_	Fe_2_O_3_	CaO	MgO	SO_3_
Fly ash/%	48.76	16.22	23.91	2.05	1.44	0.89
Slag/%	32.02	10.19	1.31	40.99	9.33	1.82
Clinker/%	21.46	4.44	4.69	64.69	2.89	0.25
Gypsum/%	5.68	1.48	1.91	44.51	4.06	41.45

**Table 4:** Fly ash composite cementitious material cementitious strength test program.

Number	Fly Ash/%	Cement Clinker/%	Desulfurization Gypsum/%	Experimental Design Ideas
A1	40	10	8	L9(3^3^) orthogonal test
A2	45	15	8	
A3	50	20	8	
A4	50	15	10	
A5	45	10	10	
A6	40	20	10	
A7	40	15	12	
A8	45	20	12	
A9	50	10	12	
B1	40	8	10	Less clinker and more gypsum
B2		8	12	
B3		8	14	
B4		10	10	
B5		10	12	
B6		10	14	
B7		12	10	
B8		12	12	
B9		12	14	
C1	45	10	8	More clinker and less gypsum
C2		10	10	
C3		10	12	
C4		12	8	
C5		12	10	
C6		12	12	
C7		14	8	
C8		14	10	
C9		14	12	

## Data Availability

All data, models, and code generated or used during the study appear in the submitted article.
